# Discussions on the Paper of Dr. G. M. Saliba, Roanoke, Ala.

**Published:** 1910-10

**Authors:** 


### DISCUSSIONS ON THE PAPER OF DR. G. M. SALIBA, ROANOKE, ALA.

### "INTESTINAL EXCESSIVE ACTIVITY PHYSIOLOGICAL CONSIDERATION."

Dr. W. H. Moon, Goodwater, Alabama.

I want to thank the doctor, and congratulate him on that excellent paper, for we know it was an excellent one, it is a subject in which we are all interested. I remember that during the early years of my practice, when I went to see a child with bowel trouble, every one would say, "Doctor you must scarify the gums," and as that seemed the thing to do, I did it. Finally, I begun to realize that grown people had this diarrhoea as well as babes, and I began to advise diet, cool clothing, cleanliness etc., and got good results, having a great deal less of this diarhea in teething children. Since we begun the prophylactic treatment, we are having a great deal less trouble with both grown people and babies along this line.

Dr. Geo. E. Pettey, Memphis, Tenn.

We have so few papers on internal medicine, I fear the surgeons will take the meetings, and the internes are to blame, they will not talk. Too many children die, entirely too many; they are not being properly studied. His position on too frequent feeding is well taken, fasting is excellent treatment; fasting not only for one day, but for several days, and you will have less cause to use other remedies. Castor Oil is a sheet anchor, it goes through the tube and takes out the poison, does not deplete to the degree that any other purgative does. Colonic flushings are fine, as most of the germs are in the colon. The salt solction is good, also muriate quinine is fine. A growing child.is often given more to eat before it has been allowed time to digest a former feeding. If the child's stomach is allowed to become empty before another feeding, it would be beneficial.

Dr. A. L. Harlan, Alexander City, Alabama.

I am an internal medicine man. I wish I could discuss this ecellent paper intelligently, xor add something to it, but cannot do so. I want to say that one of the difficulties I meet in my practice along this line outside of getting the mothers to believe that teething does not cause this diarhea, is to keep them from over feeding the child. One babe, one year old, taken suddenly with gastro-intestinal trouble, came under my care. I impressed upon the parents that one of the principal causes, in my opinion, was there was some offending matter setting up irritation, and this must be removed. On this theory I absolutely cleansed the intestines by the means of old-time Castor Oil by mouth, saline enemas by rectum. After the third day the child was better, and still better on the fourth day. I told the mother, who was nursing it, if she would not feed it for a few hours more, and manage it properly, the babe would get well. I called frequently, as I was deeply interested, it being a neighbor's child, an devery-thing seemed to be getting along nicely, but I still cautioned against over feeding. I had a call to the country, was absent some little time, and the mother nursed the babe, and as soon as fermentation took place, new infection set up, and in a short while we lost the child. This is to show what doctors have to contend with in treating diarrhea in children. I have concluded that proper management of the mother and grandmother has lots to do with the recovery of the child. Colonic flushings, and proper hygiene have a very important place in the treatment of diarrhoea.

Dr. H. B. Disharoon, Roanoke, Ala.

Fasting is the main thing in this trouble, according to my idea, and the strange part of it is, that the child often has more sense about this than the grown people. In extreme vomiting it is a good idea to wash out the stomach, even of small children.

With that, and the colon flushing, and come back to the feed slowly, a great many cases can be relieved.

Dr. G. M. Saliba, in closing.

Very sorry there was not a more general discussion. In my experience as a physician, I have found that when the digestive organs are disarranged, you have trouble to deal with. The digestive organs are a problem that confronts the medical profession, and we must study them. People object to some methods because methods are extreme, but in my practice if the people did not accept my practice, they must send for another doctor. As to the fact of fasting, it is nothing more than a natural method, if you are guided by, and follow nature in getting rid of offensive matter in the stomach, you empty it and get rid of it. There is danger in bacteria. I close with one appeal, pay more attention to diagnosis, and do not let the laity get the opinion that the doctor is wokingr for nothing but the dollar.

